# LVEF-based phenotyping in very old adults hospitalised for heart failure: cardiological discrimination, shared geriatric burden, and treatment gaps

**DOI:** 10.3389/fcvm.2026.1942320

**Published:** 2026-09-01

**Authors:** Rémi Esser, Sophie Balesdent, Jennifer Corny, Marine Larbaneix, Alejandro Mondragon, Marlène Esteban, Christine Farges, Marc Harboun, Sophie Nisse Durgeat, Vincenzo Palermo, Olivier Maurou

**Affiliations:** 1 Department of Cardiogeriatrics, Hôpital La Porte Verte, Versailles, France; 2 Department of Pharmacy, Hôpital La Porte Verte, Versailles, France; 3 Medical Affairs, NP Medical, Bordeaux, France; 4 Cardiology Department, Centre Cardiologique Marie Lannelongue, Le Plessis Robinson, France

**Keywords:** cardiogeriatric phenotyping, geriatric assessment, heart failure, iron deficiency, LVEF phenotype, treatment gap, very old adults

## Abstract

**Methods:**

We conducted a retrospective observational cohort study in a cardiogeriatric unit to compare cardiological, geriatric, biological, and therapeutic profiles across LVEF phenotypes in very old adults hospitalised for heart failure (HF). All patients with HF and classifiable LVEF were included and categorised according to 2021 European Society of Cardiology (ESC) criteria as heart failure with reduced ejection fraction (HFrEF, ≤40%), heart failure with mildly reduced ejection fraction (HFmrEF, 41%–49%), and heart failure with preserved ejection fraction (HFpEF, ≥50%). The primary objective was to describe and compare cardiological, biological, geriatric, and therapeutic profiles across LVEF phenotypes. The comprehensive geriatric assessment (CGA)-derived geriatric indicator count was used as a descriptive measure of multidimensional geriatric burden, and not as a validated frailty instrument.

**Results:**

Among 1,299 patients [median age 88.5 years (IQR 82.5–92.2)], HFpEF predominated (58.8%). Despite markedly different cardiological profiles, CGA-derived geriatric burden was similar across phenotypes: the study-defined geriatric indicator count was identical in all groups [median 2 (1–3); *p* = 0.915; *η*²=0.00014]. Iron deficiency affected 52.8% of patients, with no significant difference across phenotypes (*p* = 0.782). Clinically significant anaemia with concomitant iron deficiency was more frequent in HFpEF than in HFrEF (16.1% vs. 9.5%; *p* = 0.001), whereas clinically significant anaemia without iron deficiency was similar across groups (*p* = 0.986). In HFrEF, 52.6% received ≥3/4 guideline-directed therapeutic pillars (defined as ACEi/ARB/ARNi, beta-blocker, MRA, and SGLT2i). Overall ACEi/ARB/ARNi pillar coverage was 53.2% in HFrEF, but ARNi uptake and MRA prescription declined with increasing age, with ARNi uptake falling from 44% before age 85 to 21% after age 90.

**Conclusions:**

LVEF category remains central to pharmacological treatment selection but does not discriminate CGA-derived geriatric burden or iron deficiency burden in very old HF patients. These findings support systematic comprehensive geriatric assessment and iron status assessment regardless of LVEF phenotype, alongside phenotype-informed pharmacological optimisation.

## Introduction

Heart failure (HF) is one of the leading causes of hospitalisation and death in adults aged ≥75 years (1). In this age group, HF with preserved ejection fraction (HFpEF) predominates, driven by the high prevalence of hypertension, obesity, and atrial fibrillation (AF/flutter) (2). The 2021 European Society of Cardiology (ESC) Heart Failure Guidelines formalised three phenotypes based on left ventricular ejection fraction (LVEF): HF with reduced ejection fraction (HFrEF) (≤40%), HF with mildly reduced ejection fraction (HFmrEF) (41%–49%), and HFpEF (≥50%), each with distinct pathophysiology and treatment recommendations (3).

Very old patients with HF present a clinical profile that differs substantially from trial populations: the median age in landmark trials (PARADIGM-HF, EMPEROR-Reduced, DAPA-HF) was 63–67 years (4, 5). Geriatric syndromes — malnutrition, cognitive impairment, functional dependency, depressive syndrome, and social isolation — are prevalent in this population and may drive outcomes independently of cardiac function (6). Comprehensive geriatric assessment (CGA) provides a multidimensional framework to identify these domains in older adults with complex cardiovascular disease. A critical and underexplored question is which dimensions of the cardiogeriatric profile are discriminated by LVEF category in very old patients, and which dimensions — including CGA-derived geriatric burden — are shared across conventional LVEF-based phenotypes. From a geriatric medicine perspective, determining whether conventional cardiological phenotypes also identify patients with higher CGA-derived geriatric burden is clinically important, because access to CGA, nutritional assessment, cognitive evaluation, and structured post-discharge care should not depend solely on cardiac phenotype.

Iron deficiency affects 30%–50% of ambulatory and up to 70% of hospitalised HF patients (7). ESC guidelines recommend intravenous iron in symptomatic iron-deficient patients with HFrEF/HFmrEF to improve symptoms, exercise capacity, and quality of life, with potential effects on HF hospitalisation risk (3). In IRONMAN and HEART-FID, numerical reductions in cardiovascular outcomes were observed, although neither trial met its primary hierarchical endpoint, and the magnitude of benefit remains uncertain (8, 9). Data on iron deficiency prevalence across LVEF phenotypes in very old real-world patients remain scarce.

Despite guideline-directed quadruple therapy being recommended in HFrEF without an upper age limit, higher age is consistently associated with underuse in observational registries (3, 10). Available evidence from subgroup analyses and *post hoc* studies suggests preserved relative benefit of foundational therapies in older adults, although patients aged ≥85 years with high geriatric burden remain underrepresented in pivotal trials (11). The magnitude of this treatment gap in patients with a median age approaching 90 years, and which drug classes are most affected, have not been specifically characterised.

In this study, we performed an integrated real-world phenotypic comparison of HFrEF, HFmrEF, and HFpEF in a large cohort of very old adults hospitalised in a specialised cardiogeriatric HF unit. Rather than testing LVEF as a surrogate for frailty, our aim was to determine which dimensions of care are discriminated by LVEF phenotype and which are shared across phenotypes. We therefore compared cardiological, biological, CGA-derived geriatric, and therapeutic profiles across LVEF categories; identified variables independently associated with LVEF phenotype and admission NT-proBNP; characterised iron deficiency and clinically significant anaemia patterns; and described age-related differences in guideline-directed pharmacological treatment among patients with HFrEF.

## Methods

### Study design and setting

We conducted a retrospective, observational, single-centre cohort study at the cardiogeriatric unit of Hôpital La Porte Verte (Versailles, France). This specialised unit provides integrated cardiogeriatric care: systematic transthoracic echocardiography, NT-proBNP measurement at admission and discharge, and structured CGA for all hospitalised patients. Data were extracted from the institutional prospective database on 13 May 2026. All analyses are restricted to the index hospitalisation; no post-discharge follow-up data are available. The study was reported per STROBE and RECORD guidelines (12, 13).

Patients were admitted to the cardiogeriatric unit according to local hospital pathways for older adults with acute or decompensated HF requiring combined cardiological and geriatric management. The unit does not represent an unselected HF ward population, but a specialised inpatient cardiogeriatric setting. Accordingly, the cohort should be interpreted as representative of very old HF patients referred for integrated cardiogeriatric care rather than of all older adults hospitalised with HF.

### Inclusion and exclusion criteria

Eligible patients were all consecutive adults hospitalised in the cardiogeriatric unit during the study period with a discharge diagnosis of heart failure and available LVEF allowing classification into HFrEF, HFmrEF, or HFpEF. Patients were included irrespective of age, comorbidity burden, geriatric status, or treatment pattern. Patients without a heart failure discharge diagnosis were excluded, as were patients with unclassifiable LVEF. All analyses were performed at the hospitalisation level and restricted to the index hospitalisation recorded in the institutional database.

### Study population and definitions

All consecutive patients with a discharge diagnosis of HF and classifiable LVEF were included. HF was diagnosed per 2021 ESC Guidelines, integrating clinical features, NT-proBNP, and echocardiographic findings (3). Patients with unclassifiable LVEF (*n* = 15, 1.1%) were excluded.

The study-defined geriatric indicator count (range 0–6) summed six binary CGA-derived items: activities of daily living (ADL) dependency, malnutrition, severe cognitive impairment, depressive syndrome, social isolation, and palliative care status. ADL dependency was defined as Katz ADL score <6/6 (14). Malnutrition was defined as any documented grade of malnutrition according to routine CGA-based nutritional assessment, including Mini Nutritional Assessment-based evaluation when available (15). Severe cognitive impairment was defined as documented major neurocognitive disorder or severe cognitive impairment recorded during CGA. Depressive syndrome was defined as documented depressive symptoms, ongoing antidepressant treatment for depressive disorder, or a clinical diagnosis of depressive syndrome recorded during CGA. Social isolation was defined as living alone without reliable caregiver support, absence of an identified informal caregiver, or documented social vulnerability requiring social work intervention. Palliative care status was defined as documented palliative orientation or limitation of disease-directed escalation during hospitalisation. This composite count is descriptive, was constructed for the present analysis, and is not externally validated as a frailty index. No validated frailty scale was systematically available for the entire cohort; therefore, frailty was not analysed as a separate validated construct.

Iron deficiency was defined per the 2021 ESC Heart Failure Guidelines (3) as ferritin <100 ng/mL, or ferritin 100–299 ng/mL and transferrin saturation (TSAT) < 20%; computable in 1,274/1,299 (98.1%). Anaemia was defined as haemoglobin <10 g/dL, to capture clinically significant anaemia in the acute cardiogeriatric setting; this threshold differs from World Health Organization (WHO) diagnostic criteria and was selected to prioritise clinical relevance in hospitalised very old adults.

Guideline-directed therapy coverage in HFrEF was defined as the receipt of ≥3 of 4 foundational therapeutic pillars: renin–angiotensin system inhibition with an angiotensin-converting enzyme inhibitor (ACEi), angiotensin receptor blocker (ARB), or angiotensin receptor–neprilysin inhibitor (ARNi); beta-blocker; mineralocorticoid receptor antagonist (MRA); and sodium-glucose cotransporter-2 inhibitor (SGLT2i). ARNi prescription was additionally analysed separately as a marker of advanced renin–angiotensin system optimisation (16).

### Study endpoints

The primary objective was the structured phenotypic comparison of very old adults hospitalised for HF across LVEF categories, integrating cardiological, biological, CGA-derived geriatric, and therapeutic variables. The study-defined geriatric indicator count was analysed as a descriptive measure of multidimensional geriatric burden across LVEF phenotypes. Secondary analyses included: (i) the distribution of individual cardiological, CGA-derived geriatric, biological, and therapeutic characteristics across LVEF phenotypes; (ii) the prevalence of iron deficiency and clinically significant anaemia, including anaemia with and without concomitant iron deficiency, across LVEF phenotypes; (iii) variables independently associated with HFpEF and HFmrEF compared with HFrEF; (iv) determinants of admission log(NT-proBNP); and (v) age-related differences in guideline-directed therapeutic pillar coverage among patients with HFrEF, using ACEi/ARB/ARNi, beta-blocker, MRA, and SGLT2i as the four therapeutic pillars.

### Collected variables

Collected variables included demographic characteristics, cardiovascular history and comorbidities, echocardiographic phenotype, biological parameters, geriatric assessment items, remote monitoring status, and pharmacological treatments at discharge. Cardiovascular variables included LVEF category, ischaemic cardiomyopathy, atrial fibrillation or flutter, hypertension, diabetes, dyslipidaemia, pacemaker or implantable cardioverter-defibrillator, chronic kidney disease stage, and mitral regurgitation ≥ moderate. Biological variables included NT-proBNP at admission and discharge, haemoglobin, ferritin, transferrin saturation, albumin, and renal function. Geriatric variables included ADL dependency, malnutrition, severe cognitive impairment, depressive syndrome, social isolation, and palliative care status. Treatment variables included renin–angiotensin system inhibitors (ACEi/ARB/ARNi), beta-blocker, MRA, SGLT2i, diuretic, oral anticoagulant, total number of prescribed medications, and the presence of polypharmacy, defined as ≥10 medications. Drug-specific contraindications, intolerance, prior failed titration, detailed blood pressure-related reasons for non-prescription, hyperkalaemia episodes, and reasons for non-prescription were not systematically available. Remote monitoring status referred to enrolment in the institutional HF remote monitoring programme at discharge or during the care pathway, involving structured symptom and weight surveillance with clinical review by the cardiogeriatric team according to local practice.

### Statistical analysis

Continuous variables were inspected for distributional shape using histograms and quantile–quantile plots. Because most continuous variables were non-normally distributed and because the study was primarily descriptive, continuous variables are reported as median [IQR] and compared using Kruskal–Wallis tests. Categorical variables are reported as *n* (%) and compared using chi-squared tests. Effect sizes [η²≈H/(n−1); Cramér's V] were systematically reported to distinguish statistical from clinical significance. Missing data were handled by complete-case analysis for each specific analysis. Denominators are reported whenever data were not available for the full cohort. No multiple imputation was performed, given the descriptive and exploratory nature of the study and the low proportion of missingness for most key variables. The extent of missing data was reported for major variables when relevant.

Multivariable logistic regression identified variables independently associated with HFpEF vs. HFrEF (*n* = 1,146; Nagelkerke R² = 0.31) and HFmrEF vs. HFrEF (*n* = 533; EPV = 13.5 — borderline power, results interpreted cautiously) (17). Covariates were selected *a priori*: age, sex, obesity, ischaemic cardiomyopathy, atrial fibrillation or flutter (AF/flutter), hypertension, diabetes, malnutrition, Charlson Comorbidity Index (CCI), mitral regurgitation (MR) ≥ moderate, and chronic kidney disease (CKD) stage. Continuous predictors were standardised as z-scores. Results are reported as adjusted odds ratios (aORs) with 95% confidence intervals (CIs). For the analysis of guideline-directed therapy coverage among patients with HFrEF, patients were stratified into four age groups: 70–80, 80–85, 85–90, and >90 years. Trends across ordered age categories were assessed using a chi-squared test for trend.

Multivariable linear regression identified determinants of log (NT-proBNP) at admission (*n* = 1,288, R² = 0.259). CKD stage was mapped from text ordinal prior to analysis (99.7% available). A pre-specified LVEF×obesity interaction was non-significant (F = 1.48, *p* = 0.228). NT-proBNP kinetics (Δ% admission to discharge) were a pre-specified exploratory endpoint; regression-to-mean was quantified (Spearman r = –0.20 overall; r = –0.26 within HFrEF; both *p* < 0.001). Given the observational and hypothesis-generating nature of the study, no adjustment for multiple comparisons was performed. Secondary and exploratory analyses should therefore be interpreted as descriptive or hypothesis-generating, as appropriate. All tests were two-tailed, with *α* = 0.05. Analyses were performed using Python 3.12 (NumPy, pandas, SciPy, scikit-learn).

### Ethics

This retrospective, non-interventional observational study used pseudonymised routine care data from the institutional cardiogeriatric database and was classified as research not involving human participants (RNIPH) under French law. No additional procedure, visit, or patient contact was performed for research purposes. Data processing complied with the MR-004 reference methodology of the French Data Protection Authority (Commission Nationale de l'Informatique et des Libertés, CNIL) and with the General Data Protection Regulation (GDPR). Patients were informed of the secondary use of their healthcare data and of their right to object. In accordance with French regulations for this type of study, individual written informed consent was not required. The study was conducted in accordance with the Declaration of Helsinki.

## Results

### Study population

Of 1,471 hospitalisations, 1,314 (89.3%) had an HF discharge diagnosis; 1,299 (98.9%) had classifiable LVEF (Supplementary Figure S1). HFpEF predominated: 764 (58.8%), HFrEF 387 (29.8%), HFmrEF 148 (11.4%).

### Demographic and geriatric characteristics

Median age was 88.5 [82.5–92.2] years. HFpEF patients were older (89.1 vs. 86.7 years in HFrEF; *p* < 0.001), more frequently female (60.7% vs. 40.8%; *p* < 0.001), and more obese (15.7% vs. 4.9%; *p* < 0.001). Charlson CCI was identical (median 8 in all groups; *p* = 0.414) (Table 1).

**Table 1:** Demographic and geriatric characteristics overall and by LVEF category.

Variable	Overall (*n* = 1,299)	HFrEF (*n* = 387)	HFmrEF (*n* = 148)	HFpEF (*n* = 764)	*p*-value
Age (years), median [IQR]	88.5 [82.5–92.2]	86.7 [81.1–91.5]	88.5 [83.2–92.1]	89.1 [83.2–92.5]	<0.001
Female sex, *n* (%)	702 (54.0%)	158 (40.8%)	80 (54.1%)	464 (60.7%)	<0.001
BMI (kg/m²), median [IQR]	23.5 [20.5–27.4]	23.0 [19.9–26.1]	23.4 [20.6–27.0]	24.0 [20.7–28.2]	0.001
Obesity (BMI ≥30 kg/m²), *n* (%)	152 (11.7%)	19 (4.9%)	13 (8.8%)	120 (15.7%)	<0.001
Charlson CCI, median [IQR]	8.0 [7.0–9.0]	8 [(7–9)]	8 [(6–9)]	8 [(7–9)]	0.414
Malnutrition, *n* (%)	823 (63.4%)	266 (68.7%)	93 (63.3%)	464 (61.0%)	0.036
Severe cognitive impairment, *n* (%)	247 (19.0%)	76 (19.6%)	31 (20.9%)	140 (18.3%)	0.707
Depressive syndrome, *n* (%)	297 (22.9%)	83 (21.4%)	33 (22.3%)	181 (23.7%)	0.683
Palliative care, *n* (%)	298 (22.9%)	94 (24.3%)	33 (22.3%)	171 (22.4%)	0.753
Study-defined geriatric indicator count (0-6), median [IQR]	2 [(1–3)]	2 [(1–3)]	2 [(1–3)]	2 [(1–3)]	0.915
≥1 CGA-derived geriatric indicator, *n* (%)	1,163 (89.5%)	340 (87.9%)	131 (89.1%)	689 (90.5%)	0.365
≥2 CGA-derived geriatric indicators, *n* (%)	800 (61.6%)	243 (62.8%)	84 (56.8%)	473 (61.9%)	0.321
Remote monitoring, *n* (%)	879 (67.7%)	262 (67.7%)	101 (68.2%)	516 (67.5%)	0.986

Data are presented as median [interquartile range] for continuous variables and *n* (%) for categorical variables. Comparisons across LVEF categories were performed using Kruskal–Wallis tests for continuous variables and chi-squared tests for categorical variables. The geriatric indicator count is a study-defined descriptive count ranging from 0 to 6, including ADL dependency, malnutrition, severe cognitive impairment, depressive syndrome, social isolation, and palliative care status. It was constructed for descriptive purposes and is not externally validated. *P*-values refer to comparisons across HFrEF, HFmrEF, and HFpEF groups. ADL, activities of daily living; BMI, body mass index; CCI, Charlson Comorbidity Index; HFmrEF, heart failure with mildly reduced ejection fraction; HFpEF, heart failure with preserved ejection fraction; HFrEF, heart failure with reduced ejection fraction; IQR, interquartile range; LVEF, left ventricular ejection fraction.

The study-defined geriatric indicator count did not differ across LVEF phenotypes. The study-defined geriatric indicator count was identical in all three groups [median 2 (1–3) in each; *p* = 0.915; *η*² = 0.00014 — negligible]. More than 87% of patients in every phenotype had at least one CGA-derived geriatric indicator (*p* = 0.365), and 57%–63% had two or more (all *p* > 0.35). The ADL score differed statistically across groups (*p* = 0.0002), with a negligible effect size (*η*²<0.002) and identical median values. Malnutrition showed a borderline gradient (HFrEF 68.7% > HFpEF 61.0%; *p* = 0.036) but with *η*²<0.001. Remote monitoring enrolment was similar across phenotypes: 67.7% in HFrEF, 68.2% in HFmrEF, and 67.5% in HFpEF (*p* = 0.986).

### Cardiovascular profile, biology, and iron status

Ischaemic cardiomyopathy was the most discriminating variable: 56.3% in HFrEF, 43.9% in HFmrEF, and 26.6% in HFpEF (*p* < 0.001). Dyslipidaemia showed a significant inverse gradient (74.2%→66.2%→57.2%; *p* < 0.001). Pacemaker/ICD was more prevalent in HFrEF (32.3% vs. 16.9%; *p* < 0.001). CKD stage ≥3 was more common in HFrEF (84.8% vs. 78.8%; *p* = 0.014). AF/flutter, hypertension, and diabetes did not differ significantly. NT-proBNP at admission differed markedly across phenotypes, with median values of 6,724, 4,440, and 2,856 pg/mL in HFrEF, HFmrEF, and HFpEF, respectively (*p* < 0.001). Median NT-proBNP values in HFmrEF were between those observed in HFrEF and HFpEF (Table 2).

**Table 2:** Cardiovascular profile, biological parameters, and iron status overall and by LVEF category.

Variable	Overall	HFrEF	HFmrEF	HFpEF	*p*-value
	(*n* = 1,299)	(*n* = 387)	(*n* = 148)	(*n* = 764)	
Ischaemic cardiomyopathy, *n* (%)	486 (37.4%)	218 (56.3%)	65 (43.9%)	203 (26.6%)	<0.001
AF/flutter, *n* (%)	941 (72.4%)	269 (69.5%)	115 (77.7%)	557 (72.9%)	0.150
Hypertension, *n* (%)	871 (67.1%)	245 (63.3%)	102 (68.9%)	524 (68.6%)	0.173
Pacemaker/ICD, *n* (%)	293 (22.6%)	125 (32.3%)	40 (27.0%)	129 (16.9%)	<0.001
CKD stage ≥3, *n* (%)	1,038 (80.2%)	328 (84.8%)	111 (75.0%)	599 (78.8%)	0.014
NT-proBNP admission (pg/mL), median [IQR]	4,035 [1,587–9,062]	6,724 [3,281–15,972]	4,440 [1,884–8,526]	2,856 [1,259–6,384]	<0.001
Albumin (g/L), median [IQR]	32.7 [29.4–36.0]	33.0 [29.7–36.0]	33.0 [29.2–36.1]	32.5 [29.2–35.8]	0.593
Anaemia (Hb <10 g/dL), *n* (%)	284/1,297 (21.9%)	70/386 (18.1%)	25/148 (16.9%)	189/763 (24.8%)	0.011
Iron deficiency, *n* (%)	673/1,274 (52.8%)	204/380 (53.7%)	73/144 (50.7%)	396/750 (52.8%)	0.782
→ Anaemia + iron deficiency	169/1,274 (13.3%)	36/380 (9.5%)	12/144 (8.3%)	121/750 (16.1%)	0.001
→ Anaemia without iron deficiency	110/1,274 (8.6%)	33/380 (8.7%)	12/144 (8.3%)	65/750 (8.7%)	0.986
TSAT (%), median [IQR]	15.0 [10.0–22.0]	17.0 [11.9–23.4]	15.0 [10.0–21.0]	14.2 [10.0–21.0]	0.0004
MR ≥ moderate, *n* (%)	280/1,298 (21.6%)	116/386 (30.1%)	38/148 (25.7%)	126/764 (16.5%)	<0.001

Data are presented as median [interquartile range] for continuous variables and *n* (%) for categorical variables. Comparisons across LVEF categories were performed using Kruskal–Wallis tests for continuous variables and chi-squared tests for categorical variables. Iron deficiency was defined according to the 2021 ESC heart failure guideline definition as ferritin <100 ng/mL, or ferritin 100–299 ng/mL with TSAT <20%. Anaemia was defined as haemoglobin <10 g/dL. *P*-values refer to comparisons across HFrEF, HFmrEF, and HFpEF groups. AF, atrial fibrillation; CKD, chronic kidney disease; ESC, European Society of Cardiology; Hb, haemoglobin; HFmrEF, heart failure with mildly reduced ejection fraction; HFpEF, heart failure with preserved ejection fraction; HFrEF, heart failure with reduced ejection fraction; ICD, implantable cardioverter-defibrillator; IQR, interquartile range; LVEF, left ventricular ejection fraction; MR, mitral regurgitation; NT-proBNP, N-terminal pro-B-type natriuretic peptide; TSAT, transferrin saturation.

#### Iron deficiency and anaemia

Anaemia (Hb <10 g/dL) was more prevalent in HFpEF (24.8% vs. 18.1% in HFrEF; *p* = 0.011). Iron deficiency (ESC 2021 definition) was present in 52.8% of the cohort and did not differ significantly across phenotypes: 53.7% (HFrEF), 50.7% (HFmrEF), 52.8% (HFpEF; *p* = 0.782). Clinically significant anaemia with concomitant iron deficiency was more frequent in HFpEF (16.1% vs. 9.5% in HFrEF; *p* = 0.001), whereas clinically significant anaemia without iron deficiency was similar across all phenotypes (8.7% in HFrEF, 8.3% in HFmrEF, 8.7% in HFpEF; *p* = 0.986).

### Pharmacological treatments and guideline therapy coverage in HFrEF

**Figure 1:**
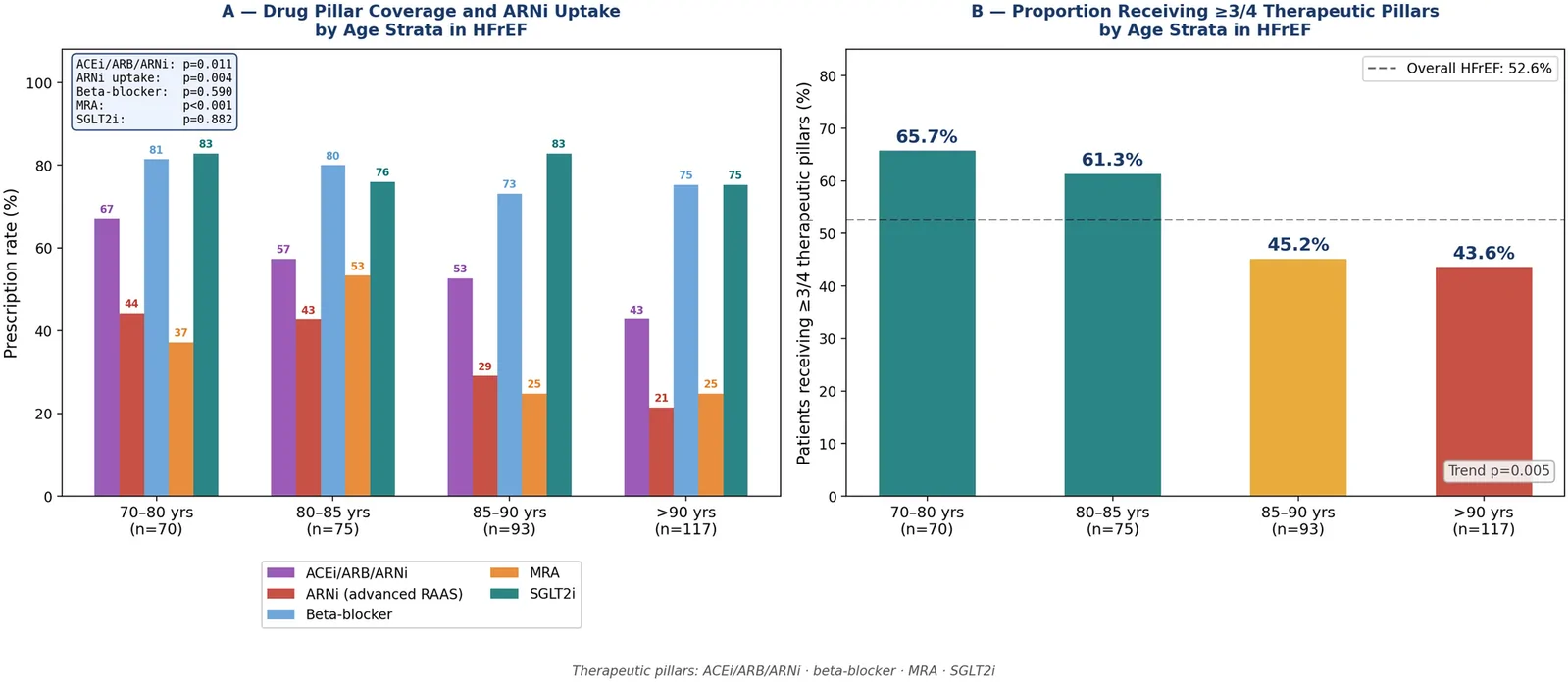
Age-dependent guideline-directed therapeutic pillar coverage and ARNi uptake in patients with HFrEF. Left panel: prescription rates of individual therapeutic pillars and ARNi uptake by age strata in patients with HFrEF. The renin–angiotensin system inhibitor pillar was defined as any prescription of ACEi, ARB, or ARNi. ARNi is shown separately as a marker of advanced renin–angiotensin system optimisation. Right panel: proportion of patients receiving at least 3 of 4 therapeutic pillars by age strata (trend *p* = 0.005). *P*-values shown for individual drugs correspond to chi-squared tests for trend across ordered age categories. ARNi uptake *p* = 0.004; ACEi/ARB/ARNi pillar *p* = 0.011; MRA *p* < 0.001; SGLT2i *p* = 0.882; beta-blocker *p* = 0.590. ACEi, angiotensin-converting enzyme inhibitor; ARB, angiotensin receptor blocker; ARNi, angiotensin receptor–neprilysin inhibitor; HFrEF, heart failure with reduced ejection fraction; MRA, mineralocorticoid receptor antagonist; SGLT2i, sodium-glucose cotransporter-2 inhibitor.

Among the HFrEF patients with available discharge treatment data, guideline-directed therapeutic pillar coverage was defined using four pillars: ACEi/ARB/ARNi, beta-blocker, MRA, and SGLT2i. Overall, 52.6% of HFrEF patients received ≥3/4 therapeutic pillars. ACEi/ARB/ARNi pillar coverage was 53.2% in HFrEF; of these, 32.1% received ARNi specifically, analysed separately as a marker of advanced renin–angiotensin system optimisation. Beta-blocker and SGLT2i were the most frequently prescribed drug classes (77.0% and 78.9%, respectively), whereas MRA coverage was lower (34.1%). Polypharmacy was prevalent: 34.3% of HFrEF patients received ≥10 medications simultaneously Figure 1, (Table 3; Supplementary Table S1).

**Table 3:** Pharmacological treatments at discharge overall and by LVEF phenotype.

Drug	Overall (*n* = 1,223)	HFrEF (*n* = 361)	HFmrEF (*n* = 143)	HFpEF (*n* = 719)	*p*-value
Medications (all classes), median [IQR]	8.0 [6.0–9.5]	8 [7–10]	8 [7–9.5]	7 [6–9]	<0.001
≥10 medications, *n* (%)	306 (25.0%)	124 (34.3%)	36 (25.2%)	146 (20.3%)	<0.001
ACEi/ARB/ARNi, *n* (%)^a^	475 (38.8%)	192 (53.2%)	75 (52.4%)	208 (28.9%)	<0.001
ARNi (sacubitril/valsartan), *n* (%)	157 (12.8%)	116 (32.1%)	29 (20.3%)	12 (1.7%)	<0.001
ACEi/ARB, *n* (%)	321 (26.2%)	77 (21.3%)	46 (32.2%)	198 (27.5%)	0.021
Beta-blocker, *n* (%)	786 (64.3%)	278 (77.0%)	107 (74.8%)	401 (55.8%)	<0.001
MRA, *n* (%)	246 (20.1%)	123 (34.1%)	31 (21.7%)	92 (12.8%)	<0.001
SGLT2i, *n* (%)	1,012 (82.7%)	285 (78.9%)	122 (85.3%)	605 (84.1%)	0.071
Diuretic, *n* (%)	1,122 (91.7%)	326 (90.3%)	133 (93.0%)	663 (92.2%)	0.473
Oral anticoagulant, *n* (%)	878 (71.8%)	247 (68.4%)	112 (78.3%)	519 (72.2%)	0.078
≥3/4 therapeutic pillars — HFrEF^b^	—	190/361 (52.6%)	—	—	—

Data are median [IQR] or *n* (%). Comparisons across LVEF categories by Kruskal–Wallis (continuous) and chi-squared (categorical) tests.

a ACEi/ARB/ARNi, any renin–angiotensin system inhibitor (ACEi, ARB, or ARNi); reported as a single pillar. ARNi and ACEi/ARB shown separately below.

b Guideline-directed therapeutic pillar coverage assessed only in HFrEF; defined as receipt of ≥3 of 4 foundational pillars: ACEi/ARB/ARNi, beta-blocker, MRA, SGLT2i. ARNi is shown separately as a marker of advanced renin–angiotensin system optimisation. ACEi, angiotensin-converting enzyme inhibitor; ARB, angiotensin receptor blocker; ARNi, angiotensin receptor–neprilysin inhibitor; MRA, mineralocorticoid receptor antagonist; SGLT2i, sodium-glucose cotransporter-2 inhibitor.

Age-stratified analyses showed a decline in ≥3/4 therapeutic pillar coverage after age 85, from 65.7–61.3% before age 85 to 45.2–43.6% after age 85 (trend *p* = 0.005). ACEi/ARB/ARNi pillar coverage also declined with age [67.1% [70–80 years] to 42.7% [>90 years]; *p* = 0.011]. This age-related decline in the RAAS inhibitor pillar was accompanied by lower ARNi uptake — falling from 44.3–42.7% before age 85 to 29.0% (85–90 years) and 21.4% (>90 years; *p* = 0.004) — while ACEi/ARB coverage also varied across age categories. MRA prescription also declined markedly (37.1–53.3% before age 85 vs. 24.7–24.8% after; *p* < 0.001), while SGLT2i and beta-blocker use remained relatively stable (*p* = 0.882 and *p* = 0.590, respectively).

### Variables independently associated with LVEF phenotype

**Figure 2:**
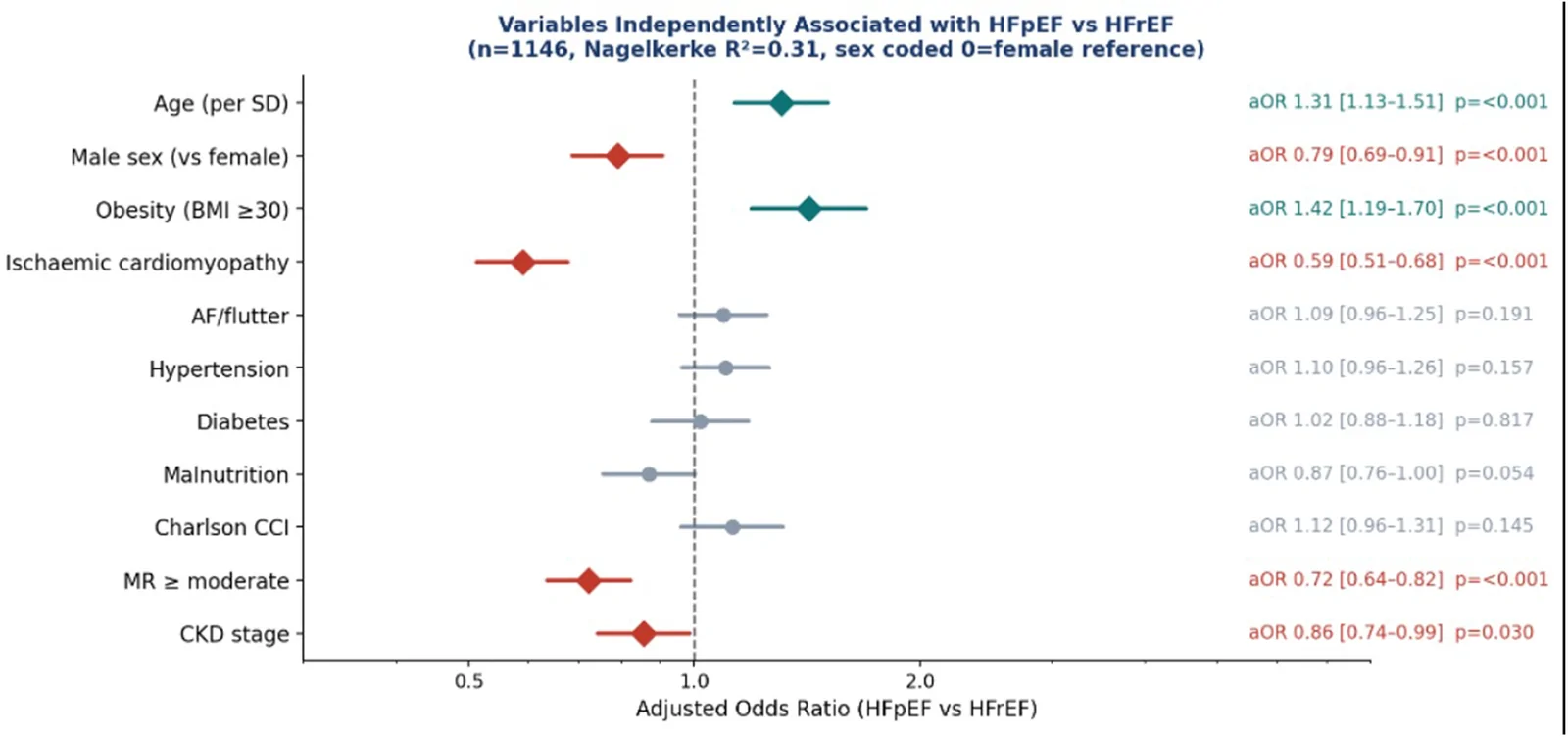
Variables independently associated with HFpEF versus HFrEF. Forest plot showing adjusted odds ratios and 95% confidence intervals from the multivariable logistic regression model comparing HFpEF with HFrEF. The model included age, sex, obesity, ischaemic cardiomyopathy, AF/flutter, hypertension, diabetes, malnutrition, Charlson CCI, MR ≥ moderate, and CKD stage. Age was analysed per standard deviation increase, and CKD stage was analysed as an ordinal variable. Adjusted odds ratios greater than 1 indicate higher odds of HFpEF compared with HFrEF, whereas adjusted odds ratios lower than 1 indicate lower odds of HFpEF compared with HFrEF. The vertical dashed line represents an adjusted odds ratio of 1. The model included 1,146 patients and had a Nagelkerke R² of 0.31. AF, atrial fibrillation; aOR, adjusted odds ratio; BMI, body mass index; CCI, Charlson Comorbidity Index; CI, confidence interval; CKD, chronic kidney disease; HFpEF, heart failure with preserved ejection fraction; HFrEF, heart failure with reduced ejection fraction; MR, mitral regurgitation; SD, standard deviation.

In the multivariable logistic model (HFpEF vs. HFrEF, *n* = 1,146), six variables were independently associated with HFpEF phenotype. Continuous predictors were standardised before modelling; therefore, the reported odds ratio for age reflects a one-standard-deviation increase rather than a one-year increase. CKD stage was analysed as an ordinal variable. Male sex was independently and negatively associated with HFpEF [aOR 0.79 (0.69–0.91); *p* < 0.001]. Additional independently associated variables: older age [aOR 1.31 (1.13–1.51); *p* < 0.001], obesity [aOR 1.42 (1.19–1.70); *p* < 0.001], absence of ischaemic cardiomyopathy [aOR 0.59 (0.51–0.68); *p* < 0.001], absence of MR ≥ moderate [aOR 0.72 (0.64–0.82); *p* < 0.001], and lower CKD stage [aOR 0.86 per stage (0.74–0.99); *p* = 0.030]. Nagelkerke R² = 0.31 (Figure 2).

For HFmrEF vs. HFrEF (*n* = 533; EPV = 13.5 — borderline power), only age was independently associated with phenotype [aOR 1.28 (1.02–1.61); *p* = 0.031]. In the descriptive analysis, HFmrEF showed intermediate values for ischaemia, obesity, female sex, and NT-proBNP. The model had borderline power (EPV = 13.5).

The multivariable linear regression (*n* = 1,288, R² = 0.259) identified LVEF category, CKD stage (*β* = +0.255/stage; *p* < 0.001; +29.1% NT-proBNP per stage), BMI (*β*=-0.055/kg/m²; *p* < 0.001; −5.4% per kg/m²), AF/flutter, Charlson CCI, and age as independently associated with log (NT-proBNP). The LVEF×obesity interaction was non-significant (F = 1.48, *p* = 0.228).

In the exploratory analysis of NT-proBNP kinetics, HFrEF showed a proportionally smaller decrease than the other phenotypes (−13.4% vs. −26.2% and −24.8%; *p* = 0.0004). This difference remained statistically significant in the subgroup of non-palliative patients (−17.5% vs. −26.5%; *p* = 0.002). Regression-to-mean was observed (Spearman r = −0.20 overall; r = −0.26 within HFrEF; both *p* < 0.001).

## Discussion

**Figure 3:**
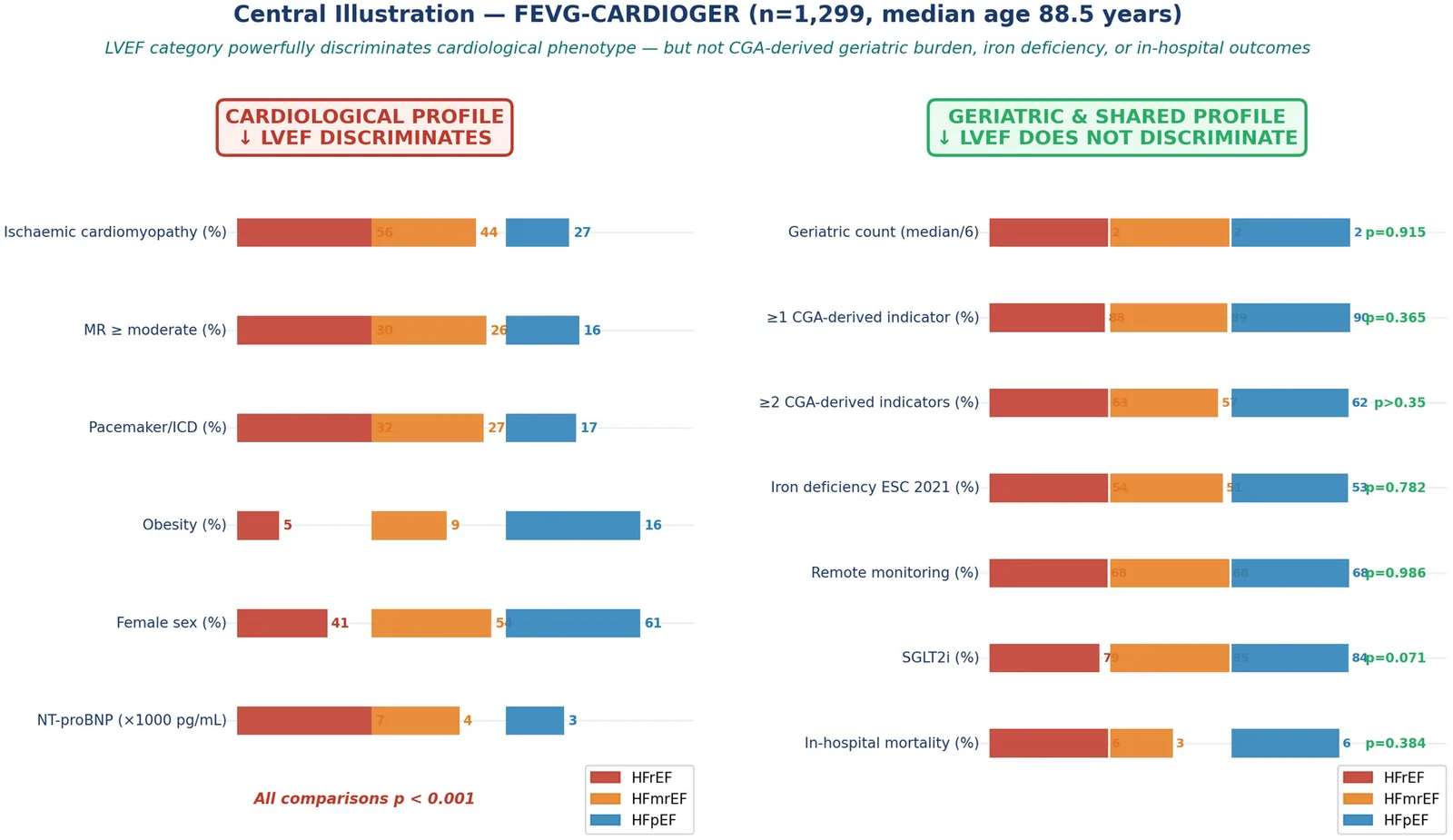
Central illustration: LVEF discriminates cardiological phenotype but not CGA-derived geriatric burden or shared geriatric characteristics. The left panel shows variables that differed markedly across LVEF categories, including ischaemic cardiomyopathy, MR ≥ moderate, pacemaker/ICD, obesity, female sex, and admission NT-proBNP. The right panel shows variables that were similarly distributed across LVEF categories or showed similar distributions across LVEF categories, including CGA-derived geriatric burden, iron deficiency, remote monitoring, SGLT2i prescription, and in-hospital mortality. The figure summarises variables showing marked differences or similar distributions across LVEF categories. *P*-values refer to comparisons across HFrEF, HFmrEF, and HFpEF groups. ESC, European Society of Cardiology; HFmrEF, heart failure with mildly reduced ejection fraction; HFpEF, heart failure with preserved ejection fraction; HFrEF, heart failure with reduced ejection fraction; ICD, implantable cardioverter-defibrillator; LVEF, left ventricular ejection fraction; MR, mitral regurgitation; NT-proBNP, N-terminal pro-B-type natriuretic peptide; SGLT2i, sodium-glucose cotransporter-2 inhibitor.

This large real-world cardiogeriatric cohort of 1,299 very old patients (median age 88.5 years) provides four main findings (Figure 3). First, LVEF category did not meaningfully discriminate CGA-derived geriatric burden, and conversely the study-defined geriatric indicator count does not meaningfully separate LVEF phenotypes — supporting a phenotype-independent framework for cardiogeriatric care. Second, iron deficiency is a frequent and phenotype-independent finding that warrants systematic screening. Third, the pattern of excess iron-deficient anaemia in HFpEF suggests a distinct, though speculative, pathophysiological mechanism. Fourth, a substantial guideline therapy gap in HFrEF worsens markedly after age 85, driven by lower ARNi uptake and MRA prescription.

The higher NT-proBNP concentrations observed in HFrEF should be interpreted in light of phenotype-related differences in chronic kidney disease and obesity, as both conditions independently influence natriuretic peptide concentrations. In our cohort, CKD stage ≥3 was more frequent in HFrEF, whereas obesity was more prevalent in HFpEF, potentially contributing to the observed NT-proBNP gradient across LVEF phenotypes. These findings highlight that NT-proBNP differences between phenotypes should be interpreted within the broader clinical context rather than attributed solely to LVEF category.

The observed differences in NT-proBNP kinetics should be considered exploratory. Because regression-to-mean was observed and longitudinal biomarker trajectories were not available, these findings cannot establish phenotype-specific biological mechanisms.

### CGA-derived geriatric burden is shared across LVEF phenotypes: implications for cardiogeriatric care

The finding that >87% of patients in every LVEF category had at least one CGA-derived geriatric indicator, with a composite count of 2 and *η*² = 0.00014, challenges the implicit assumption that HFpEF — older, more female, more metabolic — carries greater geriatric burden than HFrEF. This is not merely a lack of statistical power: the effect size was very small, suggesting limited between-group variation in this cohort. Our finding is consistent with prior studies showing that frailty in HF transcends cardiac phenotype (6) and supports systematic CGA for all hospitalised HF patients regardless of LVEF. The uniform implementation of remote monitoring across LVEF phenotypes in our unit (67.7–68.2%, *p* = 0.986) provides a practical example that phenotype-independent care pathways are feasible in very old HF patients. Extending this same organisational logic to systematic CGA may therefore represent a realistic and actionable strategy, rather than a purely theoretical recommendation.

In this context, specialised heart failure nursing and multidisciplinary follow-up models may represent key components of therapeutic optimisation in complex older patients. Recent real-world data suggest that specialised heart failure nursing consultations can improve uptake of guideline-directed therapies in HFrEF, supporting the role of structured multidisciplinary pathways in this population (18).

The higher prevalence of pacemaker or ICD implantation in HFrEF may reflect both the higher burden of structural heart disease and a greater prevalence of conduction disorders in this phenotype. In some patients, chronic right ventricular pacing may also contribute to or aggravate left ventricular systolic dysfunction through pacing-induced dyssynchrony. Because pacing burden, device indication, lead position, and longitudinal LVEF trajectory were not available, this mechanism cannot be tested in the present dataset and should be interpreted as a hypothesis-generating explanation.

### Iron deficiency: a frequent and phenotype-independent finding

Iron deficiency was present in 52.8% of patients and was similarly distributed across all three LVEF phenotypes (*p* = 0.782). This phenotype-independent pattern — affecting more than one in two very old hospitalised HF patients — supports systematic iron status assessment at admission regardless of LVEF. The ESC 2021 guidelines give a Class I recommendation for intravenous iron in iron-deficient symptomatic HFrEF, based on trials demonstrating symptomatic and functional benefit (3, 19). Both IRONMAN and HEART-FID reported numerical differences in cardiovascular outcomes, although neither trial met its primary hierarchical endpoint and the extent of benefit across HF phenotypes remains uncertain (8, 9). Whether the benefit extends fully to very old patients with high geriatric burden requires prospective confirmation in this specific population.

Anaemia with concomitant iron deficiency was more frequent in HFpEF (16.1% vs. 9.5%; *p* = 0.001), while anaemia without iron deficiency was similar across phenotypes (8.7%, *p* = 0.986). This pattern may reflect cardiometabolic inflammation and hepcidin-mediated iron restriction in the context of greater obesity prevalence in HFpEF (15.7% vs. 4.9%) (20, 21), although this mechanism remains speculative in the absence of inflammatory markers or hepcidin measurements in this dataset.

### The age-dependent guideline therapy gap in HFrEF: prescribing complexity in very old adults

The approximately 20 percentage-point decline in receipt of ≥3/4 guideline-directed therapeutic pillars after age 85 (from 65.7–61.3% to 45.2–43.6%; trend *p* = 0.005) is a clinically important finding, but should not be interpreted as inappropriate underuse in all cases. When ACEi, ARB, and ARNi were grouped within a single renin–angiotensin system inhibitor pillar, ACEi/ARB/ARNi coverage also declined with age (from 67.1% at 70–80 years to 42.7% at >90 years; *p* = 0.011), indicating that age-related differences affected the RAAS inhibitor pillar as a whole, not only ARNi uptake. However, the decline in ARNi uptake was particularly steep — falling from 44% before age 85 to 21% after age 90 (*p* = 0.004) — suggesting that age-related prescribing differences are most pronounced for advanced renin–angiotensin system optimisation and for MRA, while SGLT2i and beta-blocker use remained relatively stable (11).

In very old adults, pharmacological optimisation is shaped by multimorbidity, renal dysfunction, hypotension, hyperkalaemia risk, polypharmacy, frailty, treatment goals, and tolerance during acute hospitalisation (3, 10, 11). This drug-specific pattern is clinically plausible: ARNi initiation requires blood pressure tolerance and titration, while MRA use requires careful renal and potassium monitoring in a population with frequent CKD (3, 11). In contrast, SGLT2i require no titration, have a low risk of hyperkalaemia, and may be easier to implement in complex older patients (11).

These findings therefore do not simply indicate a treatment gap, but rather highlight the need for structured, individualised prescribing support in cardiogeriatric care. In patients aged ≥85 years, optimisation of HFrEF therapy should combine guideline awareness with systematic assessment of renal function, blood pressure, electrolyte risk, frailty, polypharmacy, patient priorities, and expected benefit (11, 16, 22). Future interventions should test whether cardiogeriatric medication review can improve appropriate use of disease-modifying therapies while maintaining safety in very old adults.

### HFmrEF as a clinically relevant transition zone

Although the HFmrEF subgroup was smaller and the multivariable model had borderline power, this phenotype should not be dismissed as merely intermediate. In our cohort, HFmrEF showed a transitional cardiological profile, with intermediate rates of ischaemic cardiomyopathy, obesity, female sex, and NT-proBNP levels, consistent with the recognised heterogeneity of HFmrEF (3, 23). More importantly, HFmrEF showed a treatment pattern closer to HFrEF than to HFpEF for several selected guideline-relevant therapies: beta-blockers were prescribed in 74.8% of HFmrEF patients and ACEi/ARB/ARNi coverage was 52.4%, whereas ARNi uptake and MRA use were lower, at 20.3% and 21.7%, respectively. This distinction is important because overall RAAS inhibitor pillar coverage should be interpreted separately from ARNi uptake alone. This suggests that very old patients with HFmrEF may share part of the same therapeutic optimisation challenge as those with HFrEF, particularly for drug classes requiring careful blood pressure, renal function, and potassium monitoring (11, 22). Given the smaller sample size and the absence of treatment tolerance data, these findings should be interpreted as exploratory, but they support including HFmrEF patients in future cardiogeriatric prescribing-support strategies rather than treating them as a neutral intermediate category.

## Limitations

This is a retrospective, single-centre, in-hospital study. The absence of post-discharge follow-up is the principal limitation: in-hospital mortality (5%–6%) is a poor surrogate for prognosis in very old HF patients, where most adverse events occur after discharge. The reasons for non-prescription of ARNi and MRA — including hypotension, hyperkalaemia, severity of renal dysfunction, prior intolerance, patient preference, and therapeutic limitation decisions — were not systematically available in this dataset and may partly explain the observed treatment gap beyond age itself. NT-proBNP kinetics are exploratory; regression-to-mean cannot be excluded. The HFmrEF model had borderline power (EPV = 13.5). The study-defined geriatric indicator count is descriptive and not externally validated; no validated frailty scale was systematically available for the whole cohort, and cognitive impairment was recorded as a binary severe impairment indicator rather than through a harmonised neuropsychological score. Because the study was primarily designed to compare phenotypic distributions rather than to develop predictive models, the geriatric indicator count was not evaluated as an independent determinant of LVEF phenotype or treatment allocation. Anaemia was defined as haemoglobin <10 g/dL to capture clinically significant anaemia in the acute cardiogeriatric setting, rather than according to WHO diagnostic thresholds. No sensitivity analysis using WHO anaemia thresholds was performed; therefore, findings related to anaemia apply specifically to clinically significant anaemia as defined in this study. The pathophysiological explanation for excess iron-deficient anaemia in HFpEF is speculative in the absence of inflammatory or hepcidin measurements. Single-centre design limits generalisability. Because this cohort was drawn from a dedicated cardiogeriatric unit, selection bias is likely. The high prevalence of CGA-derived geriatric indicators should therefore not be extrapolated to all older adults with HF, particularly those managed in conventional cardiology, internal medicine, or outpatient settings. Given the retrospective design, the present findings should be considered hypothesis-generating rather than evidence of causality or evidence of effectiveness of a specific care model. Prospective studies comparing older and very old HF populations, with standardised CGA instruments, longitudinal outcomes, and treatment tolerance data, are needed to confirm whether phenotype-independent cardiogeriatric assessment and structured prescribing support improve clinical outcomes.

## Conclusions

In very old adults hospitalised for heart failure, LVEF category remains central for cardiological phenotyping and pharmacological treatment selection, but it does not meaningfully separate CGA-derived geriatric burden or iron deficiency burden; conversely, CGA-derived geriatric burden does not meaningfully discriminate LVEF phenotype. This distinction provides the unifying message of the present study: LVEF may organise treatment algorithms, but it should not be used to restrict access to shared cardiogeriatric care needs such as systematic CGA, iron status assessment, and structured follow-up. Iron deficiency was frequent across all LVEF phenotypes, supporting systematic screening, while the clinically significant anaemia with concomitant iron deficiency in HFpEF suggests a phenotype-specific biological pattern that warrants further investigation. In parallel, age-related differences in HFrEF pharmacological treatment patterns were observed, with lower ≥3/4 therapeutic pillar coverage, ARNi uptake, and MRA prescription after age 85, despite relatively stable SGLT2i and beta-blocker use. Low ARNi uptake and MRA prescription were also observed in HFmrEF. Together, these findings support a phenotype-informed approach to pharmacological optimisation, but a phenotype-independent approach to cardiogeriatric assessment and follow-up in very old HF patients.

## Data Availability

The datasets presented in this article are not readily available because the data underlying this article cannot be shared publicly due to institutional and regulatory restrictions related to pseudonymised healthcare data. Reasonable requests may be considered by the corresponding author, subject to institutional approval and applicable data protection regulations. Requests to access the datasets should be directed to remi.esser@lpv.univi.fr.

## Supplementary material

Supplementary Table S1 Data are presented as percentages among patients with available discharge treatment data in each age stratum. Column headings indicate the number of patients with available treatment data over the total number of HFrEF patients in the corresponding age stratum. Trends across ordered age categories were assessed using the chi-squared test for trend. Guideline-directed therapeutic pillar coverage was defined as receipt of ≥3 of 4 foundational therapeutic pillars: ACEi/ARB/ARNi, beta-blocker, MRA, and SGLT2i. ARNi is shown separately as a marker of advanced renin–angiotensin system optimisation. ACEi, angiotensin-converting enzyme inhibitor; ARB, angiotensin receptor blocker; ARNi, angiotensin receptor-neprilysin inhibitor; MRA, mineralocorticoid receptor antagonist; SGLT2i, sodium-glucose cotransporter-2 inhibitor.
