# Supplementary material for: LVEF-based phenotyping in very old adults hospitalised for heart failure: cardiological discrimination, shared geriatric burden, and treatment gaps
Source: Front Cardiovasc Med. 2026 Sep 1;13:1942320. doi: 10.3389/fcvm.2026.1942320 (PMC13575673; doi:10.3389/fcvm.2026.1942320)
Supplement: Supplementary file 1 [file Table1.docx]

**Supplementary Table S1. Guideline-directed therapy coverage in patients with HFrEF aged ≥70 years by age strata.**

| **Drug** | **70–80 yrs (n=70/77)** | **80–85 yrs (n=75/80)** | **85–90 yrs (n=93/102)** | **>90 yrs (n=117/122)** | **p (trend)** |
| --- | --- | --- | --- | --- | --- |
| ACEi/ARB/ARNi | 67.1% | 57.3% | 52.7% | 42.7% | 0.011 |
| ARNi | 44.3% | 42.7% | 29.0% | 21.4% | 0.004 |
| Beta-blocker | 81.4% | 80.0% | 73.1% | 75.2% | 0.590 |
| MRA | 37.1% | 53.3% | 24.7% | 24.8% | <0.001 |
| SGLT2i | 82.9% | 76.0% | 82.8% | 75.2% | 0.882 |
| ≥3/4 therapeutic pillars | 65.7% | 61.3% | 45.2% | 43.6% | 0.005 |

Data are presented as percentages among patients with available discharge treatment data in each age stratum. Column headings indicate the number of patients with available treatment data over the total number of HFrEF patients in the corresponding age stratum. Trends across ordered age categories were assessed using the chi-squared test for trend. Guideline-directed therapeutic pillar coverage was defined as receipt of ≥3 of 4 foundational therapeutic pillars: ACEi/ARB/ARNi, beta-blocker, MRA, and SGLT2i. ARNi is shown separately as a marker of advanced renin–angiotensin system optimisation. ACEi = angiotensin-converting enzyme inhibitor; ARB = angiotensin receptor blocker; ARNi = angiotensin receptor–neprilysin inhibitor; MRA = mineralocorticoid receptor antagonist; SGLT2i = sodium-glucose cotransporter-2 inhibitor.
